# Influenza virus infection exacerbates gene expression related to neurocognitive dysfunction in brains of old mice

**DOI:** 10.1186/s12979-024-00447-y

**Published:** 2024-06-21

**Authors:** Wenxin Wu, Jeremy S. Alexander, J. Leland Booth, Craig A. Miller, Jordan P. Metcalf, Douglas A. Drevets

**Affiliations:** 1 Pulmonary, Critical Care & Sleep Medicine, Department of Medicine, University of Oklahoma Health Sciences Center, Room 425, RP1 800 N. Research Pkwy, Oklahoma City, OK 73104 USA; 2 Department of Veterinary Pathobiology, College of Veterinary Medicine, Oklahoma State University, Oklahoma State University, Stillwater, OK USA; 3 Veterans Affairs Medical Center, Oklahoma City, OK USA; 4 Department of Microbiology and Immunology, University of Oklahoma Health Sciences Center, Oklahoma City, OK USA; 5 Infectious Diseases, Department of Medicine, University of Oklahoma Health Sciences Center, 800 Stanton L. Young, Suite 7300, Oklahoma City, OK 73104 USA

**Keywords:** Influenza virus, Aging, Brain, Lung, CNS, Neuroinflammation, Cognition, Interferon

## Abstract

**Background:**

Age > 65 years is a key risk factor for poor outcomes after human influenza infection. Specifically, in addition to respiratory disease, non-neurotropic influenza A virus (IAV) causes neuro-cognitive complications, e.g. new onset depression and increases the risk of dementia after hospitalization. This study aimed to identify potential mechanisms of these effects by determining differences between young and old mice in brain gene expression in a mouse model of non-neurotropic IAV infection.

**Methods:**

Young (12 weeks) and old (70 weeks) C57Bl/6J mice were inoculated intranasally with 200 PFU H1N1 A/PR/34/8 (PR8) or sterile PBS (mock). Gene expression in lung and brain was measured by qRT-PCR and normalized to β-actin. Findings were confirmed using the nCounter Mouse Neuroinflammation Array (NanoString) and analyzed with nSolver 4.0 and Ingenuity Pathway Analysis (IPA, Qiagen).

**Results:**

IAV PR8 did not invade the central nervous system. Young and old mice differed significantly in brain gene expression at baseline and during non-neurotropic IAV infection. Expression of brain Ifnl, Irf7, and Tnf mRNAs was upregulated over baseline control at 3 days post-infection (p.i.) only in young mice, but old mice expressed more Ifnl than young mice 7 days p.i. Gene arrays showed down-regulation of the Epigenetic Regulation, Insulin Signaling, and Neurons and Neurotransmission pathways in old mice 3 days p.i. while young mice demonstrated no change or induction of these pathways at the same time point. IPA revealed marked baseline differences between old and young mice. Gene expression related to Cognitive Impairment, Memory Deficits and Learning worsened in old mice relative to young mice during IAV infection. Aged mice demonstrate more severe changes in gene expression related to memory loss and cognitive dysfunction by IPA.

**Conclusions:**

These data suggest the genes and pathways related to learning and cognitive performance that were worse at baseline in old mice were further worsened by IAV infection, similar to old patients. Early events in the brain triggered by IAV infection portend downstream neurocognitive pathology in old adults.

**Supplementary Information:**

The online version contains supplementary material available at 10.1186/s12979-024-00447-y.

## Background

Influenza is a common and important global viral pathogen that inflicts significantly higher hospitalization and mortality rates in adults > 65 years of age compared with younger adults [1–3]. Although influenza is primarily thought of as a respiratory disease, infection with influenza A virus (IAV) also impacts the central nervous system (CNS), causing a wide range of neuro-cognitive complications in humans of all ages [4–7]. The least common and most severe complications are caused by neurotropic influenza strains, mostly avian influenza, that invade the CNS [8]. Less severe sequelae of IAV infection are much more prevalent. The most common is sickness behavior, which although mild and short-lived, has an outsized economic impact due to large numbers of infected workers being absent or working while ill [9, 10].

However, more harmful neuro-cognitive issues with a delayed onset and longer duration have also been identified. For example, individuals with IAV infection are at increased risk of new onset of depression, the magnitude of which peaks 30–179 days after the index case but remains statistically elevated more than 1 year after infection [11]. In addition, IAV, as well as a variety of other infections severe enough to require hospitalization are associated with an increased risk of dementia over the ensuing 4–20 years manifested as a decline in cognition significant enough to cause impairments in daily functioning [12–14]. Given the high prevalence of IAV infection in the elderly, including severe infection, it is important to understand the mechanisms by which it causes or exacerbates neurological disorders.

Neurologic dysfunction can occur following infection with either neurotropic influenza strains that invade the CNS, or non-neurotrophic strains that lack this capacity [8]. Therefore, particularly in non-neuroinvasive infections, activation of the peripheral innate immune system triggering inflammatory cytokine production in the brain is a fundamental cause of neuro-cognitive pathologies [15, 16]. In mouse models of non-neurotropic IAV infection, there is increased expression of mRNA for the pro-inflammatory cytokines IFN-α, IL-6, TNF-α, and IL-1β in the hippocampus, as well as increased concentrations of TNF-α [17, 18]. These cytokines activate microglia which subsequently disrupt homeostatic neuronal morphology and synaptic function [17–20]. This neuro-inflammatory cascade has negative effects on cognitive functions in adult mice such as memory formation and spatial learning [17, 18].

Thus, compelling evidence indicates that aging increases morbidity and mortality during influenza infection, yet how the brains of aged animals differ in their response to IAV from those of younger animals is not well understood [21, 22]. Mouse models reveal that the inflammatory response to influenza infection is dysregulated in aging and is a fundamental cause of increased morbidity and risk of death [7, 23, 24]. In peripheral tissues, old animals exhibit a delayed specific immune response to IAV infection as well as prolonged inflammation and excess tissue damage after infection [23]. Less is known about specific alterations in the aged brain during IAV infection. Brains of aged animals have baseline alterations in immune cell composition, gene expression, and cytokine production that could be responsible for the differential responses to IAV infection observed in old as compared to young animals [25–30]. These baseline changes could be the key to causing neuroinflammation and oxidative stress that contribute to age-related cognitive impairment apart from that seen with infection [31]. Additionally, aging is also the principal risk factor for dementia and is associated with a decline in overall cognitive performance suggesting aged animals have less cognitive resilience [32, 33]. Given these unknown, yet important, features of IAV in the elderly, the goal of this current manuscript was to identify similarities and differences in gene expression in the brains of young and old mice during infection by the non-neurotropic mouse-adapted human A/PR/34/8 (PR8) H1N1 virus [34].

## Materials and methods

### Ethics Statement

The animal study was reviewed and approved by The Institutional Animal Care and Use Committee (IACUC) of the University of Oklahoma Health Sciences Center (protocol number: 17-106-HI). The facility where this research was conducted is accredited by AAALAC.

### Mice

Young (12-week) and aged (70-week) C57BL/6J male mice were purchased from Jackson Laboratories (Bar Harbor, ME) and were group housed and given food and water *ad lib*. Animals were sacrificed by overdose of isoflurane at 3-and 7-days post-infection (p.i.).

### Influenza a virus and mouse infection

The IAV strain used in this study was A/PR/34/8 (PR8). The stocks were propagated in Madin-Darby canine kidney (MDCK, ATCC and Manassas, VA) cells following standard procedures [35]. The virus was titered by plaque assay in MDCK cells, aliquots were made and stored at − 80 °C.

**Fig. 1:**
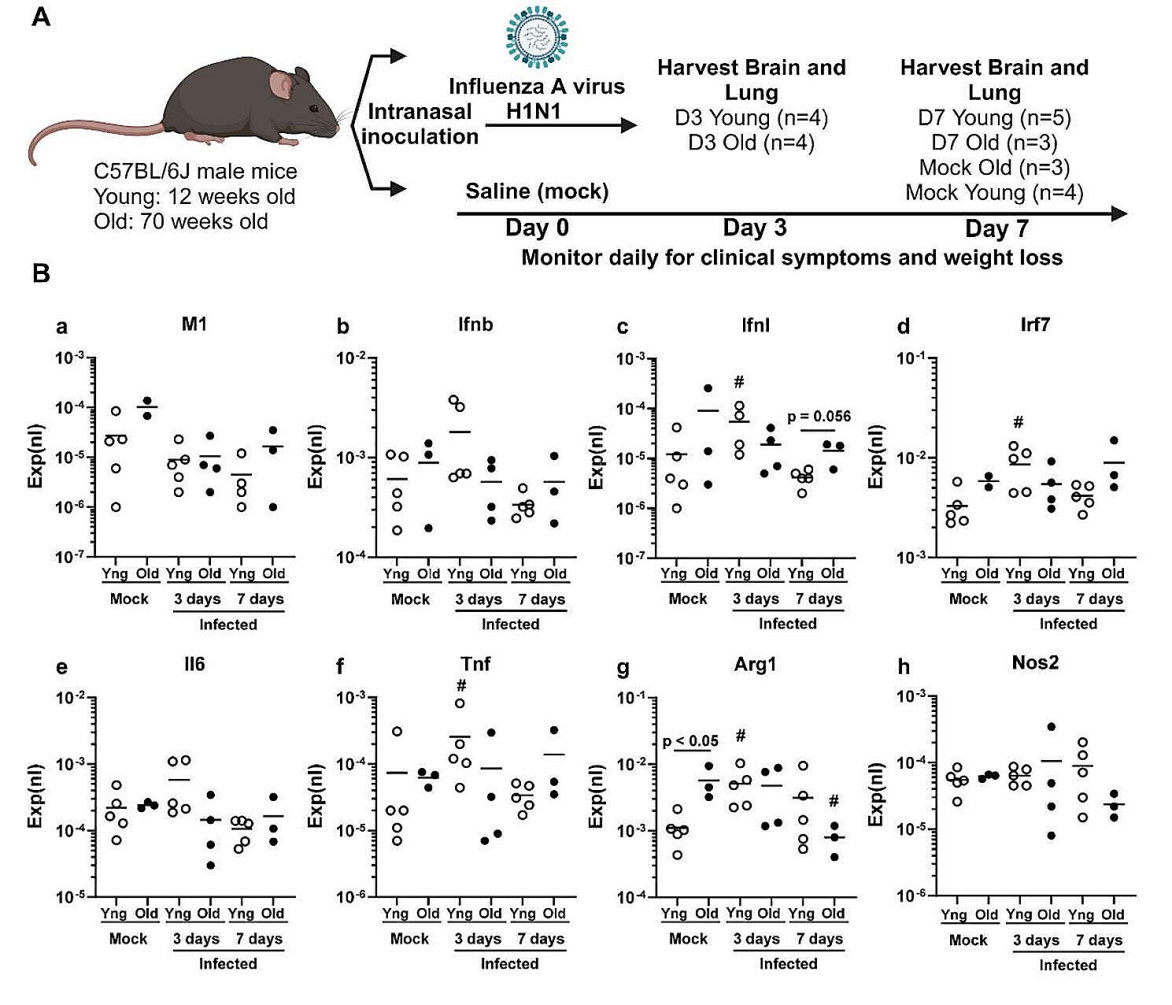
Gene expression in brains of young and old mice differs before and after non-neuroinvasive IAV infection. (**A**) Schematic of the experimental plan on IAV infection to old and young mice. The image was created with BioRender.com. (**B**) Gene expression was measured by qPCR and normalized to β-actin in brains of uninfected (mock) young and old mice, and in mice 3 and 7 days after IAV infection (Infected). Symbols represent the normalized gene expression (Exp(nl)) in individual young (○) and old (●) mice, the line represents the mean. Results in infected mice at 3 and 7 days were compared to uninfected (mock) of the same age by Kruskal-Wallis test with Benjamini, Krieger and Yekutieli post-test. Discoveries (FDR < 0.05) in infected mice versus same age mock are shown by a (#). A two-tailed Mann-Whitney test was used to compare results from different age groups at the same time point. Relevant p values are shown. Mouse numbers in each group is shown in Fig. 1A

Mice were held in a vertical position while sedated and infected by intranasal instillation of 200 plaque-forming units (PFU) PR8 virus diluted in PBS (70 µl solution). An equal volume of PBS without virus instilled intranasally was used as a control in the mock group. All infected animals were sacrificed by an overdose of isoflurane at 3- and 7-days p.i. The mock group was sacrificed at 7 days p.i. This group also served as baseline controls (Fig. 1A). Numbers of mice analyzed were as follows: young mock (*n* = 4), young day 3 (*n* = 4), young day 7 (*n* = 5), old mock (*n* = 3), old day 3 (*n* = 4), old day 7 (*n* = 3). The animals were meticulously watched both during and after each procedure to make sure they recovered properly. Mice were monitored daily for 7 days for clinical symptoms (shivering, inactivity, hunched posture, and piloerection) and their weight was recorded daily, or until the experimental endpoint, whichever came first.

### Measurement of mRNA expression by quantitative real-time PCR (qRT-PCR)

A modified TRIzol (Invitrogen, Carlsbad, CA) procedure was used to extract and quantify the total RNA from the lung and brain [36]. Electrophoresis on formaldehyde/agarose gel was used to confirm the integrity of the RNA. Using the oligo (dT) SuperScript II First-Strand Synthesis System for RT-PCR, equal amounts (1 µg) of RNA from each sample were reverse-transcribed into cDNA (Invitrogen, Carlsbad, CA). Gene specific primers’ sequences are shown in Supplemental Table 1. BDNF, NRGN, GRIN2B, SNCA, CASP1 and RALA were selected to test for their connection to brain memory cognitive function. IAV M1 was used to assess for viral replication. ifnb, ifnl, IRF7, IL6, TNFα are innate response proteins. Nos2 and Arg1 are markers for M1 and M2 macrophages respectively. qRT-PCR was carried out on a Bio-Rad CFX96TM Touch Real-Time PCR Detection System using 100 ng sample RNA and SYBR Green (Quanta Biosciences, Gaithersburg, MD). The target gene’s ΔCT value and its normalizer, β-actin, were used to calculate and plot the results.

### Histological analysis of mouse brain

Mice were euthanized at day 7 after IAV infection, and the brains were fixed in 4% paraformaldehyde in PBS for 24 h at room temperature before being embedded in paraffin. Paraffin-embedded sections were trimmed to 5 μm according to previously published methods and guidelines [37, 38] and collected onto charged slides before staining with hematoxylin and eosin (H&E) for microscopic evaluation by light microscope. Brain tissues we evaluated for: meningeal infiltrates, gliosis, perivascular edema, vascular thrombi, neuronal necrosis, and hemorrhage. All tissues were assigned a quantitative histopathological score based on previously documented criteria [39, 40]: 0 = no apparent pathology/change; 1 = minimal change (minimally increased numbers of inflammatory cells); 2 = mild change (mild inflammatory infiltrates, damage/necrosis, fibrin deposition and/or exudation); 3 = moderate change (as previously described, but more moderately extensive); 4 = marked changes (as previously described, but with severe inflammation, damage/necrosis, exudation, vasculitis and/or thrombosis). Three slices per animal were analyzed. All tissues were evaluated and scored by a board-certified veterinary pathologist (CAM) blinded to sample study group to eliminate bias and to ensure scientific rigor.

### Measurement and analysis of mRNA expression by gene array

Brains from mock-infected and IAV-infected mice 3 and 7 days p.i. were preserved in TRIzol until processed. A modified TRIzol procedure was used to extract and quantify the total RNA from the brain. Gene expression was measured in whole brains of uninfected and IAV infected young and old mice using the nCounter® Mouse Neuroinflammation Panel (NanoString Technologies, Inc.) containing probe sets for 759 genes, in addition to housekeeping genes used for normalization (Supplemental Table 2). Genes in the array are assigned to one or more functional sets based on their annotation. Results were analyzed using nSolver® 4.0 software and the Advanced Analysis 2.0 package (NanoString Technologies, Inc.). Numbers of mice analyzed were as follows: young mock (*n* = 4), young day 3 (*n* = 4), young day 7 (*n* = 5), old mock (*n* = 3), old day 3 (*n* = 4), old day 7 (*n* = 3). Significant changes in gene expression (nCounts) between uninfected (mock) and infected mice of the same age 3 or 7 days after infection, e.g. young mock versus young day 3 or old mock versus old day 3, as well as comparisons of young and old mice at the same time point, e.g. young mock versus old mock, were determined by multiple t-test with the Benjamini-Hochberg post-test, an FDR of < 0.05 was considered significant (Supplemental Tables 3 and 4). The Gene Set Analysis module was used to summarize the change in regulation within each defined gene set relative to the baseline by calculating a t-statistic for each gene against chosen covariates (nCounter Advanced Analysis 2.0 User Manual (Man-10030-03, January 2018)). In experiments reported here, results in young and old mice from days 3 and 7 were compared with uninfected (Mock) of the same age. The value calculated for a given gene set and covariate is called the global significance score and measures the cumulative evidence for the differential expression of genes in a pathway and is calculated as the square root of the mean squared t-statistic of genes. Undirected global significance scores measure the overall differential expression of the selected gene set relative to selected covariates, i.e. infection versus Mock, without regard for whether each gene is up- or down-regulated. Thus, the greater the number, the greater the change in the infected state versus Mock. The directed global significance scores are calculated similarly to undirected global significance scores, but also take into account the sign of the t-statistic. Thus, a positive score suggests up-regulation and a negative score suggests down-regulation. Additionally, gene expression data, including log2 fold-changes and Benjamini-Hochberg adjusted p values of the desired comparisons for all genes, was uploaded into Ingenuity Pathway Analysis (IPA) version 111,725,566 (Qiagen) for further analysis of canonical pathways, upstream regulators and downstream effects. IPA settings included using The Ingenuity Knowledge Base (genes only) as the reference set, “Mouse” as the species, and “Brain” for the Tissues and Cell Lines. Results are reported as Z-scores, findings of ≥ 2.0 or ≤-2.0 are considered significant. Descriptions of calculations and assumptions made in causal analytics in IPA including Upstream Regulator analysis and Downstream Effects analysis are reported in Krämer et al. [41].

### Statistical analysis

Statistical significance was determined as follows and noted in the figure legends. Briefly, p values for RT-PCR results were calculated by combining normalized gene expression values from individual young (*n* = 4–5/time point) and old (*n* = 3–4/time point) mice. Gene expression in infected mice 3 and 7 days was compared to uninfected (mock) of the same age by Kruskal-Wallis test with Benjamini, Krieger and Yekutieli post-test with a False Discovery Rate (FDR) of < 0.05 considered significant. Comparison of old and young mice at the same time point was by unpaired 2-tailed Mann-Whitney test with p values < 0.05 considered significant.

## Results

### Effect of age on brain cytokine gene expression and histopathology during IAV infection

In order to compare the innate immune responses to IAV in young vs. old mice, young (12-week) and old (70-week) C57BL/6J mice were infected intranasally (i.n.) with the IAV PR8 strain as described. The control, or mock infection, group was inoculated with an equal volume of PBS (Fig. 1A). All infected animals were sacrificed by an overdose of isoflurane at 3- and 7-days post-infection (p.i.). A detailed analysis of the effects of IAV infection on body weight, lung weight and histopathology and pulmonary gene expression is provided in an accompanying paper (Wu et al. manuscript in preparation). Briefly, we verified that infection occurred in the mice by measuring viral IAV matrix protein M1 (M1) gene expression in the lungs of young and old mice 3 and 7 days after infection. The mean normalized expression was significantly greater in aged mice (1.450 ± 1.031 (mean ± SD)) than in young mice (0.215 ± 0.229, *p* = 0.03) at day 3, after which M1 expression declined but remained detectable in both groups by day 7 and was without differences between them (data not shown). The results confirmed, as expected, that IAV M1 expression was not detected in the brain (Fig. 1B-a), as IAV PR8 is non-neurotropic. Despite the lack of neuroinvasion, peripheral IAV infection triggered a significant host response in the brain. In the initial analysis, we primarily compared the effect of IAV on cytokine responses as compared to age-matched mock-infected controls (baseline). For example, expression of interferon λ (Ifnl), interferon regulatory factor 7 (Irf7), Tumor Necrosis Factor (Tnf), and arginase 1(Arg1) were significantly increased in the brains of IAV-infected young mice 3 days p.i. compared to the mock-infected young baseline group (Fig. 1B-c, d,f, g). In contrast, none of these gene transcripts was increased in the IAV-infected old mice relative to the mock-infected old baseline group (Fig. 1B) despite their having greater IAV M1 expression in the lungs (shown in the text above). Although it appeared that interferon β (ifnb) and Il6 were induced in the brains of IAV-infected young mice 3 days p.i. compared to the mock-infected young baseline group by IAV infection of the lung, this did not reach statistical significance, and there was no apparent induction of these transcripts in the old mice (Fig. 1B-b, e). There was no apparent induction of nitric oxide synthase 2 (Nos2) in the brains of either group relative to their age-matched mock-infected baseline controls (Fig. 1B-h). When we compared the absolute expression of cytokines at day 3 in the IAV-infected young mice vs. the IAV-infected old mice, it appeared that expression of Ifnb, Ifnl, Irf7, Il6, and Tnf were higher in the young mice, vs. the old mice. On day 7, this pattern appeared to be reversed in that IAV-infected old mice had higher expression of ifnb, Ifnl, Irf7 and Tnf than in IAV-infected young mice. These results indicate that the old mice had a delayed innate response to peripheral IAV infection in the brain relative to young mice. Of interest, baseline expression of Arg1 was higher in old as compared with young mice while Ifnl was significantly greater in old than in young mice at day 7 p.i. (Fig. 1B-g, c)). Thus, preliminary analysis of gene expression confirmed that the brain responds to non-neuroinvasive IAV infection, and that young and old mice differ in both their responses to infection, as well as in baseline expression of brain cytokines.

**Fig. 2:**
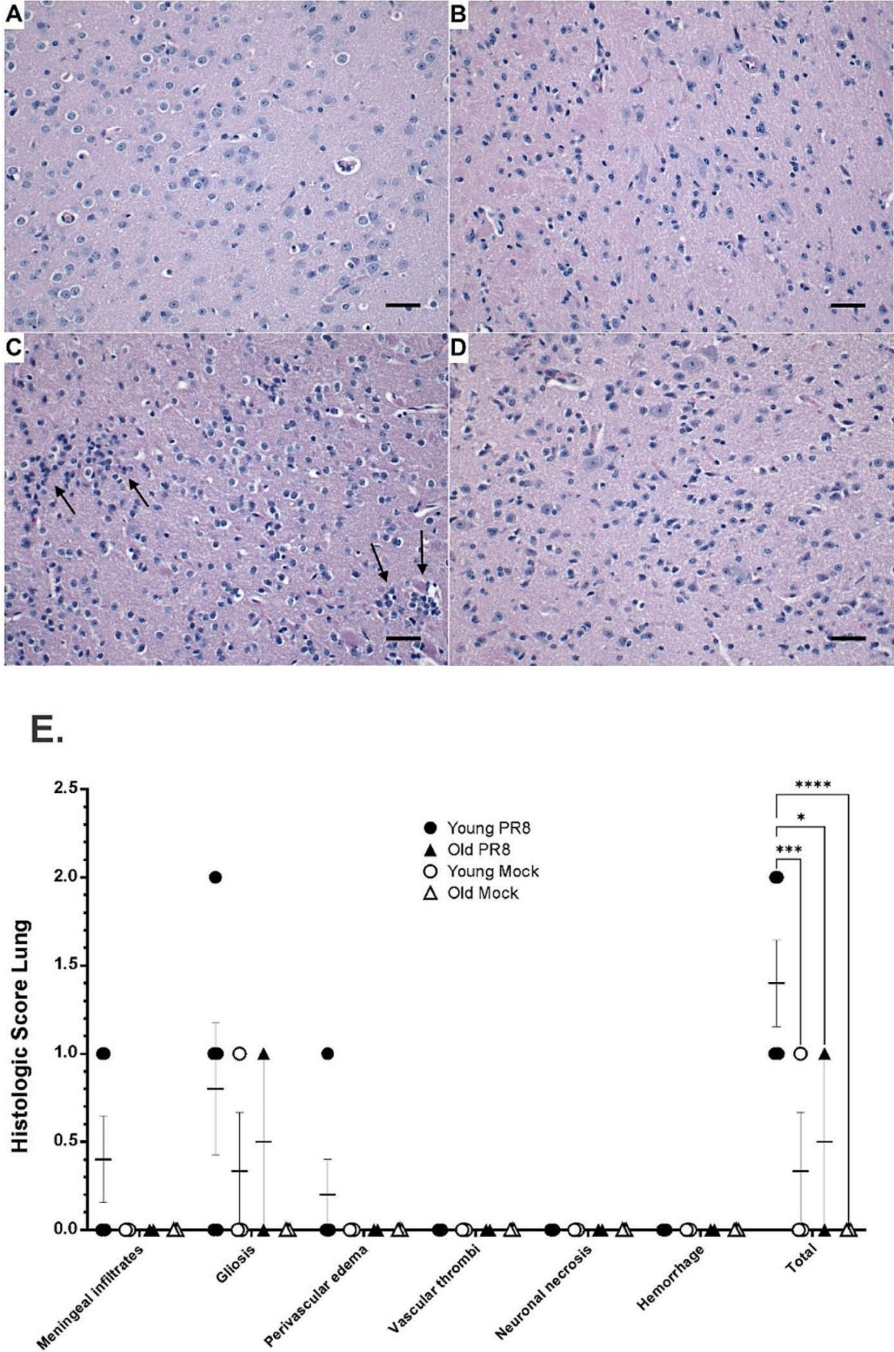
Mouse brain tissue pathology at day 7 after IAV infection. The mice were intranasally inoculated with IAV at 200 PFU/mouse. Mock treated mice were inoculated with PBS. Brain tissue was harvested at day 7 after infection. Brain tissue (mostly cerebral cortex) from both young (**A**) and old (**B**) uninfected mice was histologically normal. However, young mice with IAV infection (**C**) exhibited increased clusters of glial cells (arrows) within the neuropil and surrounding axons. Glial cells were also slightly increased in the cerebral neuropil of IAV infected old mice (**D**, arrow). Histopathologic evaluation and scoring of IAV infection were determined by a board-certified veterinary pathologist blinded to sample treatment group (**E**). Statistical analysis of histopathologic scores was performed by two-way ANOVA with Tukey’s post-test. Adjusted p values are as follows: * denotes significant difference between the two groups, *p* < 0.05. *** denotes significant difference between the two groups, *p* < 0.001. **** denotes significant difference between the two groups, *p* < 0.0001. Mouse numbers in each group are shown in Fig. 1A

Next, brains of infected young and old mice were evaluated for histopathological changes 7 days after infection. Brain tissues from control animals (Young Mock and Old Mock) were histologically unremarkable and appeared normal (Fig. 2A and B). In contrast, mononuclear inflammatory infiltrates were observed in the meninges of 2/5 young mice with IAV infection (not shown), while increased clusters of glial cells (arrows) within the neuropil and surrounding axons were observed in both young (3/5) and old mice (1/3) infected with IAV (Fig. 2C and D). Young IAV-infected mice appeared to demonstrate a slightly higher degree of gliosis than older mice with IAV infection, but this was not statistically significant (*p* = 0.200). Nonetheless, combining scores of each pathological feature showed that global infection-induced changes were significantly greater in young IAV-infected mice than in young controls. Although individual components of the histologic scores were not different between groups, when histologic scores for meningeal infiltrates, gliosis, and perivascular edema were summated (Total), infection-induced pathology also differed between the brains of young and old infected mice (Fig. 2E). In contrast, there was no significant difference in pathology scores between IAV-infected old mice when compared with their non-infected aged cohorts. Collectively, these data indicate that non-neuroinvasive IAV infection alters gene expression in the brain after lung infection and causes histopathologic changes in the brains of both young, and to a lesser extent old, animals at day 7. The difference in the histopathologic changes between old and young mice may be due to an impaired and delayed immune response in the old mice.

### Broad analysis of innate immune gene expression responses in the brain to IAV infection

The differences on brain histopathology between young and aged mice suggested larger scale changes in gene expression were likely present that might give insight into the post-infectious neurocognitive complications that follow IAV infection. Gene expression was measured in whole brains of uninfected and IAV infected young and old mice using the nCounter® Mouse Neuroinflammation Panel containing probe sets for 759 genes (Supplemental Table 2; see Methods for details).

**Fig. 3:**
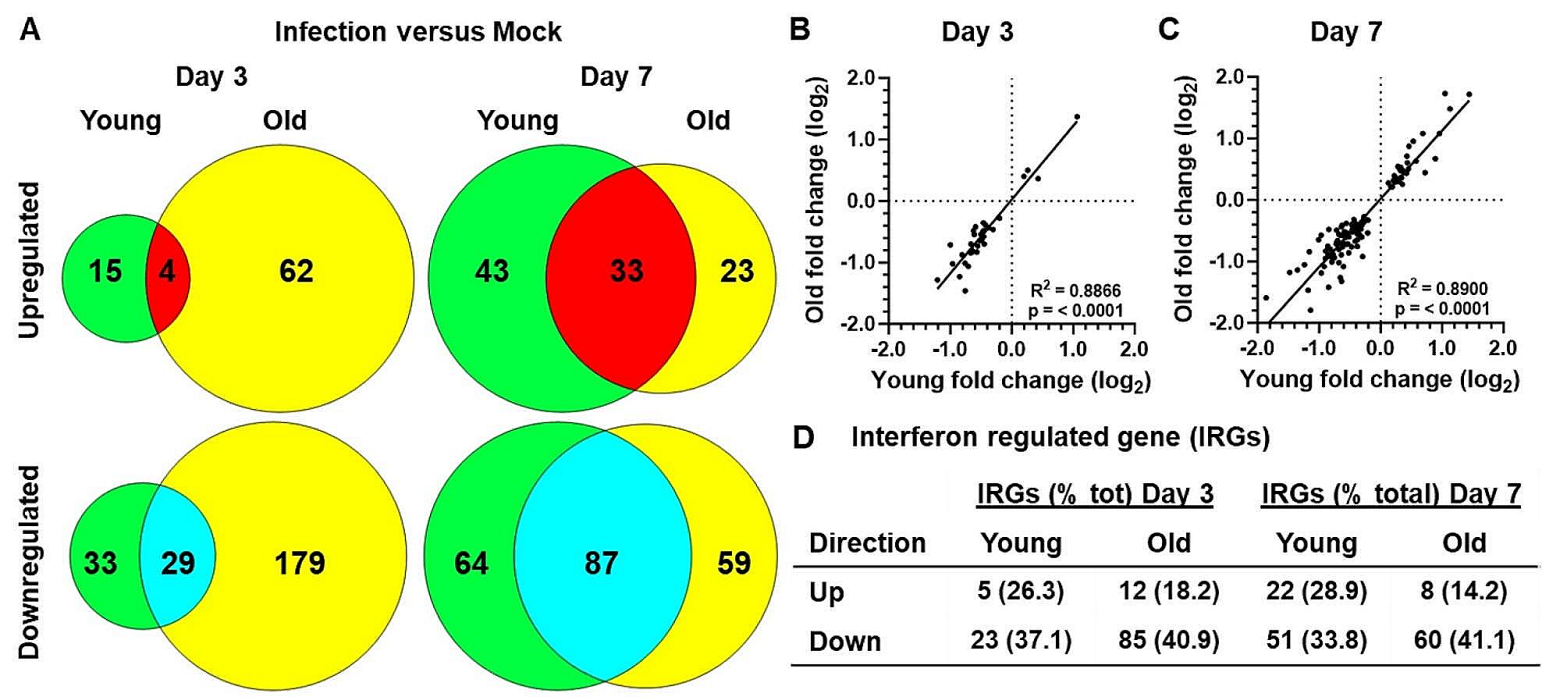
IAV infection alters gene expression in brains of young and old mice. Gene expression in brains of uninfected (mock) young and old mice, and in mice 3 and 7 days after IAV infection. Significant changes in gene expression in infected mice compared with age specific uninfected mice were determined by multiple t-test with a Benjamini-Hochberg post-test. Venn diagrams (**A**) show numbers of up- and downregulated genes 3 and 7 days after infection that were significantly changed (*p* < 0.05) in young (green) and old (yellow) mice only, as well as genes significantly changed in the same direction (up red, down blue) in both groups of mice. Scatter plots (**B**, **C**) show numbers up- and downregulated genes 3 (**B**) and 7 (**C**) days after infection that were significantly changed (*p* < 0.05) in the same direction in young and old mice. Statistical analysis of R^2^ and p values were by simple linear regression and are shown. (**D**) shows numbers (% up- or down-regulated genes) of interferon regulated genes (IRGs) as determined by the Interferome 2.01 database. Mouse numbers in each group are shown in Fig. 1A

Analysis of differentially expressed genes in infected mice compared with age-specific mock-infected cohorts showed that, in both age groups, the majority of significant changes were in genes that were down-regulated by infection at both days 3 and 7 p.i. (Fig. 3A). At day 3, there were 19 upregulated genes in young mice and 66 in old mice, 4 of which were upregulated in both groups. In contrast 62 and 208 unique genes were downregulated by infection in young and old mice, respectively, while 29 were downregulated in both age cohorts. By day 7 p.i., the numbers of unique significantly changed genes increased in young mice to 76 upregulated and 151 downregulated compared with 56 upregulated genes and 146 downregulated genes in old mice. The number of shared genes changed in the same direction in both groups also increased to 33 upregulated and 87 downregulated. Further analysis showed that genes changed in the same direction in both young and old mice did so with similar magnitudes, in aggregate, although some degree of divergence was evident (Fig. 3B and C).

Because IFN signaling is a central aspect of host defense response to IAV, we anticipated that the brain would sense and respond to IFNs produced during IAV infection [42, 43]. To test this, we enumerated the interferon regulated genes (IRGs) in mice of both ages by searching the Interferome 2.0 database with genes that showed significant up-or down-regulation compared with uninfected controls (Fig. 3D) [44]. Overall, IRGs comprised 34.6% and 32.2% of all significantly changed genes in younger mice, and 35.4% and 33.7% in old mice at days 3 and 7, respectively. Additional confirmatory evidence of these changes was confirmed by comparing expression levels of 8 genes in uninfected (mock) and day 7 infected mice measured by nCounts, with separate measurements by qRT-PCR. The results using the two methods showed a high degree of concordance between the two techniques (Supplemental Fig. 1).

There was a high degree of concordance between the most notably changed genes identified at day 3 with those also identified at day 7 (Table 1). For example, 7 of the 10 most strongly downregulated genes in young and old mice were among this group at both days 3 and 7. Interestingly, these gene changes were widespread including those involving multiple cell types in the brain including astrocytes, microglia, neurons, and oligodendrocytes, and those involving multiple functional groupings, e.g. innate and adaptive immune responses, epigenetic regulation, matrix remodeling, and lipid metabolism (Table 1). Additionally, there were several genes representing multiple different functional groupings that were significantly up- or down-regulated in infected young and old mice but were not also significantly changed at the same time point in the opposite age group (Tables 2 and 3). As found with shared genes, 8 of 17 upregulated and 5 of 18 downregulated genes in young mice were IRGs (Table 2). In contrast, only 3 of 19 genes upregulated in old mice were IRGs compared with 7 of 15 downregulated genes (Table 3).

**Table 1:** IAV infection triggers conserved changes in brain gene expression between young and old mice

Direction of change	Gene	Log_2_ fold change Day 3^1^		Log_2_ fold change Day 7^1^			Interferon regulated^2^
		Young	Old	Young		Old	
**Up-regulated**	**Kdm4a**	0.199 (0.042)	0.402 (0.083)				
	**Nlgn2**	0.260 (0.062)	0.501 (0.097)				
	**Cdkn1a**	1.060 (0.244)	1.37 (0.188)	1.44 (0.232)		1.72 (0.201)	yes
	**Eef2k**	0.429 (0.118)	0.364 (0.113)	0.721 (0.112)		0.446 (0.121)	
	**Bbc3**			0.463 (0.155)		0.875 (0.101)	
	**Esam**			0.532 (0.181)		0.955 (0.238)	
	**Fkbp5**			1.130 (0.183)		1.480 (0.145)	
	**Grm2**			0.575 (0.136)		0.631 (0.134)	
	**Islr2**			0.686 (0.235)		1.08 (0.243)	
	**Ly6a**			1.050 (0.158)		1.73 (0.206)	yes
	**Nfkbia**			0.959 (0.142)		1.080 (0.167)	yes
	**Slc2a1**			0.893 (0.132)		0.672 (0.158)	yes
**Down-regulated**	**Hpgds**	-0.664 (0.014)	-0.697 (0.152)				
	**Opalin**	-0.759 (0.126)	-1.46 (0.166)				
	**Padi2**	-0.673 (0.164)	-0.837 (0.125)				
	**Fcrls**	-1.210 (0.198)	-1.28 (0.175)	-1.86 (0.190)		-1.59 (0.187)	yes
	**Gpr34**	-0.963 (0.136)	-1.02 (0.162)	-1.18 (0.129)		-1.47 (0.175)	
	**Itgam**	-0.807 (0.137)	-0.873 (0.141)	-0.968 (0.131)		-0.573 (0.148)	
	**Olfml3**	-0.761 (0.160)	-1.010 (0.072)	-1.24 (0.153)		-1.05 (0.077)	yes
	**P2ry12**	-1.00 (0.15)	-0.713 (0.146)	-1.16 (0.142)		-0.843 (0.157)	yes
	**Pllp**	-0.849 (0.140)	-1.23 (0.126)	-1.01 (0.134)		-0.646 (0.135)	
	**Tyrobp**	-0.710 (0.148)	-1.060 (0.117)	-0.966 (0.141)		-1.19 (0.126)	yes
	**Ago4**			-1.48 (0.276)		-1.18 (0.205)	
	**Top2a**			-1.14 (0.212)		-1.79 (0.268)	
	**Ugt8a**			-1.35 (0.196)		-1.14 (0.159)	

^1^Data show the log_2_ fold change (Infection/mock) of young and old mice 3 and 7 days after infection compared with their age-specific control. Statistical analysis of gene expression was performed using multiple t-tests with the Benjamini-Hochberg method of controlling the FDR. The gene list shown is restricted to those with the 10 largest fold-changes (up or down) from their control, all have an FDR < 0.05

^2^ Interferon Regulated genes were identified by searching on the Interferome v2.01 database

**Table 2:** **Over and under expressed genes in brains of young mice after IAV infection**

Direction	Gene	Day 3^1^	Day 7^1^	IFN regulated^2^	
**Up-regulated**	Tbc1d4	0.819 (0.232)		yes	
	Kit	0.811 (0.186)			
	Casp2	0.538 (0.134)		yes	
	Slc2a1	0.504 (0.141)		yes	
	Jarid2	0.415 (0.117)			
	Hps4	0.410 (0.113)			
	Tle3	0.391 (0.067)		yes	
	Pole	1.350 (0.310)	1.640 (0.294)		
	Nostrin	0.900 (0.182)	0.970 (0.172)		
	Pecam1	0.861 (0.223)	0.910 (0.212)		
	Lcn2		3.140 (0.711)	yes	
	Tspan18		1.040 (0.368)	yes	
	Kdm4d		0.881 (0.263)		
	Ms4a4a		0.872 (0.236)		
	Ncaph		0.871 (0.283)	yes	
	Trpm4		0.830 (0.172)		
	Il1r1		0.726 (0.140)		
**Down-regulated**	Cx3cr1	-0.570 (0.118)		yes	
	Pik3cg	-0.580 (0.146)			
	Tgfa	-0.624 (0.147)			
	Mfge8	-0.628 (0.149)		yes	
	Tmem100	-0.660 (0.125)			
	Syk	-0.725 (0.185)			
	Tmem119	-0.725 (0.122)		yes	
	Top2a	-0.932 (0.223)			
	Cryba4	-0.993 (0.252)	-1.150 (0.241)		
	Slc2a5	-1.060 (0.151)	-1.260 (0.145)		
	Gjb1		-1.040 (0.211)		
	Kcnj10		-0.955 (0.235)	yes	
	Kcnk13		-0.953 (0.210)		
	Cideb		-0.915 (0.136)		
	Bcas1		-0.890 (0.169)		
	Enpp6		-0.856 (0.173)		
	Cd40		-0.840 (0.210)	yes	
	Gdpd2		-0.813 (0.269)		

^1^ Data show the log_2_ fold change (Infection/mock) of young mice 3 and 7 days after infection compared with age-specific control. Statistical analysis of gene expression was performed multiple t-tests with the Benjamini-Hochberg method of controlling the FDR. Tables show genes with the 10 greatest fold changes (up or down) from their control with an adjusted *p* < 0.05 that were significantly changed in young mice but not also in old mice

^2^ Interferon (IFN) Regulated genes were identified by searching on the Interferome v2.01 database

**Table 3:** **Over and under expressed genes in brains of old mice after IAV infection**

Direction	Gene	Day 3^1^	Day 7^1^	IFN regulated^2^	
**Up-regulated**	Fkbp5	1.260 (0.137)			
	Islr2	1.160 (0.227)			
	Bbc3	0.854 (0.094)			
	Ly6a	0.828 (0.195)		yes	
	Tbr1	0.823 (0.027)			
	Esam	0.789 (0.224)			
	Nfkbia	0.771 (0.156)		yes	
	Cldn5	0.731 (0.233)			
	E2f1	0.728 (0.158)			
	Grin2b	0.925 (0.170)	0.872 (0.182)		
	Bcl2l11		0.888 (0.249)		
	Grin2a		0.574 (0.164)		
	Suv39h1		0.570 (0.064)		
	Chst8		0.511 (0.159)		
	Jun		0.500 (0.085)		
	Abl1		0.478 (0.141)		
	Mertk		0.467 (0.080)		
	Rgl1		0.467 (0.066)		
	Rab6b		0.399 (0.077)	yes	
**Down-regulated**	Ugt8a	-1.550 (0.149)			
	Ccl2	-1.930 (0.593)		yes	
	Col6a3	-2.100 (0.406)		yes	
	Trem1	-2.400 (0.065)			
	Cxcl10	-2.800 (0.776)		yes	
	Ccr2	-1.540 (0.235)	-1.380 (0.251)		
	Chn2	-1.610 (0.471)	-1.630 (0.504)	yes	
	Tnfrsf10b	-1.680 (0.355)	-1.660 (0.383)		
	Clec7a	-2.050 (0.234)	-1.63 0(0.240)	yes	
	B3gnt5	-2.320 (0.631)	-3.690 (0.708)		
	Cytip		-2.570 (0.511)	yes	
	Sftpd		-1.910 (0.957)		
	Cd74		-1.530 (0.301)		
	Nfkb2		-1.400 (0.279)	yes	
	Btk		-1.17 0(0.333)		

^1^ Data show the log_2_ fold change (Infection/mock) of old mice 3 and 7 days after infection compared with age-specific control. Statistical analysis of gene expression was performed multiple t-tests with the Benjamini-Hochberg method of controlling the FDR. Tables show genes with the 10 greatest fold changes (up or down) from their control with an adjusted *p* < 0.05 that were significantly changed in old mice but not also in young mice

^2^ Interferon (IFN) Regulated genes were identified by searching on the Interferome v2.01 database

Direct comparisons of nCounts between young and aged mice under the same conditions, i.e. Mock, and day 3 and day 7 after IAV infection were also performed (Table 4). Differential gene expression in the uninfected mice cohorts had lower statistical significance but was consistent with previously described changes in old mice compared with young [28, 45], and the data points between the groups were clearly distinct. Moreover, 6 of the 10 discordantly regulated genes are IRGs consistent with the IFN-signature identified in brains of aged mice [46, 47]. Infection caused these differences to diminish even though expression of C4a remained significantly higher in aged mice at day 3. Ccni and Ralb were representative of genes with lower expression in aged than in young mice. Scl2a5 is downregulated in young mice compared with uninfected at days 3 and 7 p.i. as well as having significantly lower nCounts at day 7 than aged mice (Tables 2 and 4). Among differentially expressed genes identified in the old vs. young IAV-infected mice, 2 of 6 of these, both at day 3, were IRGs. Notably, B3gnt5 is downregulated among old IAV-infected animals compared to young infected animals (on day 7), and B3gnt5 is also downregulated among old infected animals compared to their mock or baseline group at day 3. This effect was increased at day 7 (Table 3). The B3gnt5 gene is associated with human disease, like salt and pepper syndrome, which is a rare autosomal recessive progressive neurological disorder.

**Table 4:** Genes differentially expressed in brains of young and old mice without and with IAV infection^1^

Comparison	Gene	Log_2_ fold change (SE)	*p* value^2^	Annotation^3^	Interferon regulated^4^
**Mock (young vs. old)**	**Bcl2a1a**	1.47 (0.156)	0.0919	Apoptosis, NF-kB	
	**Casp1**	1.46 (0.229)	0.0919	Apoptosis, Cytokine Signaling, Innate Immune Response	yes
	**Lilrb4a**	1.33 (0.199)	0.0919	Adaptive Immune Response, Inflammatory Signaling	
	**Rsad2**	1.31 (0.207)	0.0919	Inflammatory Signaling, Microglia Function	yes
	**C4a**	1.26 (0.166)	0.0919	Astrocyte Function	yes
	**Ptprc**	1.19 (0.148)	0.0919	Adaptive Immune Response, Matrix Remodeling	yes
	**Atr**	0.491 (0.067)	0.0919	Apoptosis, Cell Cycle, Cellular Stress, DNA Damage	
	**Syp**	-0.317 (0.049)	0.0919	Neurons and Neurotransmission	
	**Rab6b**	-0.421 (0.064)	0.0919	Microglia Function	yes
	**Fscn1**	-0.553 (0.088)	0.0919	Microglia Function	yes
**Day 3 (young vs. old)**	**C4a**	0.924 (0.129)	0.0762	Astrocyte Function	yes
	**Ccni**	-0.156 (0.021)	0.0762	Cell Cycle	yes
	**Ralb**	-0.217 (0.026)	0.0433	Autophagy, Growth Factor Signaling	
**Day 7 (young vs. old)**	**Slc2a5**	0.464 (0.051)	0.0298	Microglia Function	
	**Itgb5**	0.236 (0.028)	0.0298	Adaptive Immune Response, Growth Factor Signaling, Matrix Remodeling	
	**B3gnt5**	-1.350 (0.110)	0.0114	Astrocyte Function	

^1^Data show the log_2_ fold change (old/young) of young and old mice without infection (Mock) and 3 and 7 days after infection

^2^ Statistical analysis of gene expression was performed using multiple t-tests with the Benjamini-Hochberg post-test. Genes shown are those with Benjamini-Hochberg p values of < 0.1

^3^ Annotation is from the NanoString Mouse Neuroinflammation Array

^4^ Interferon Regulated genes were identified by searching on the Interferome v2.01 database

**Fig. 4:**
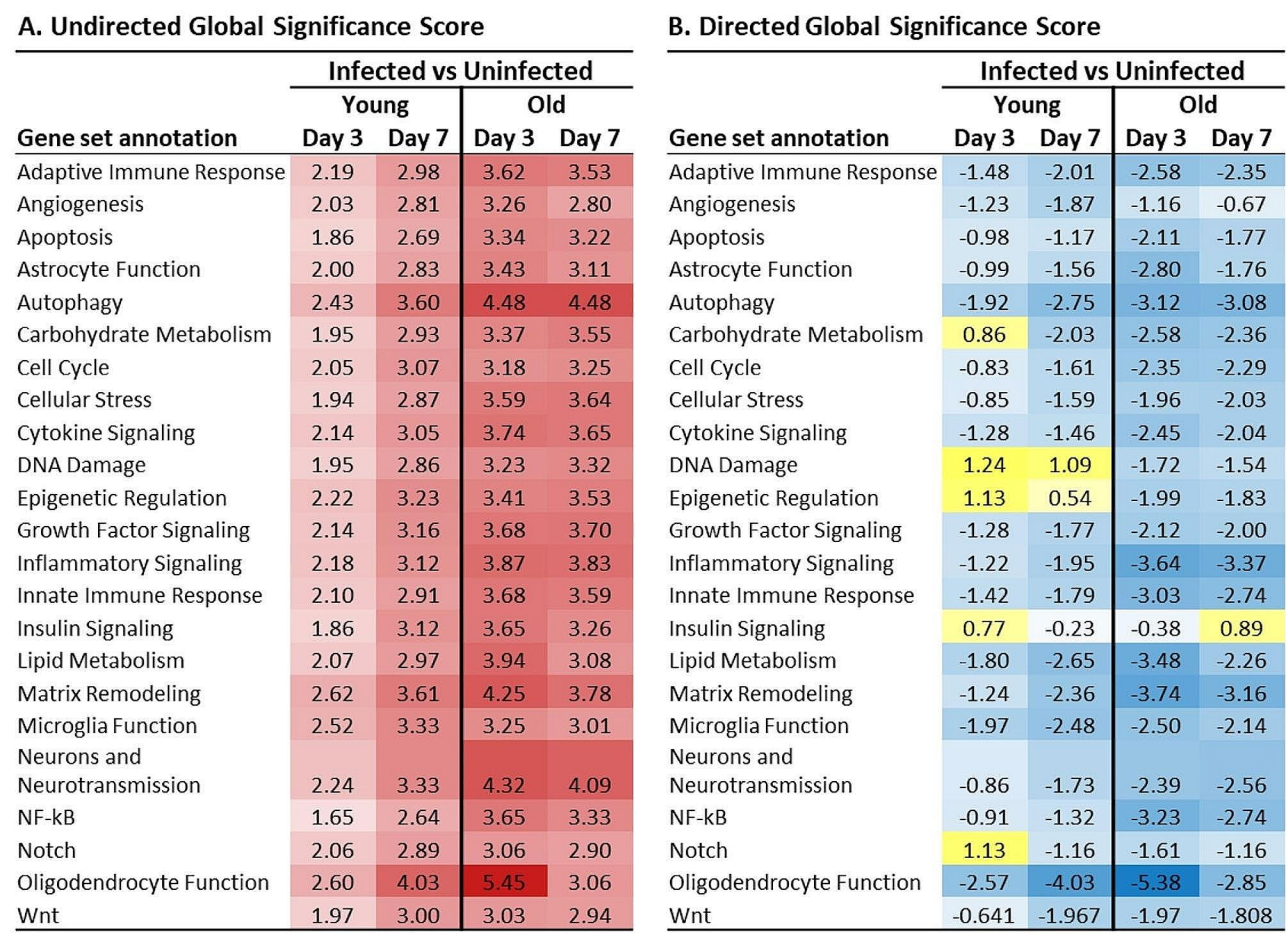
Gene set analysis shows non-neuroinvasive IAV infection triggers more robust changes in brains of old mice than in brains of young mice. Gene expression in brains was measured by nCounts in uninfected young and old mice, and in mice 3 and 7 days after IAV infection using the nCounter® Mouse Neuroinflammation panel. Results were analyzed using nSolver® 4.0 software and the Gene Set Analysis module of the Advanced Analysis 2.0 package. The value calculated for a given set is called the global significance score and measures the cumulative evidence for the differential expression of genes in a set and is calculated as the square root of the mean squared t-statistic of genes. The undirected global significance scores (**A**) measures the overall differential expression of the selected gene set in infected mice versus mock without regard for whether each gene is up- or down-regulated. Thus, the greater the number, the greater the change in set expression. The directed global significance scores (**B**) are calculated similarly, but also takes into account the sign of the t-statistic. Positive and negative scores suggest up- and down-regulation, respectively. Significance scores for gene sets from young and old mice 3 and 7 days after IAV infection are given. Heat maps were drawn in Microsoft Excel and are based on the reported score. Mouse numbers in each group are shown in Fig. 1A

Gene set analysis in nSolver summarizes the change in regulation of given sets of genes 3 and 7 days after IAV infection compared with uninfected (mock). For this, each gene on the panel is assigned membership into a particular “gene set” based on its annotation that identifies a broad biological function category. Genes in the Neuroinflammation panel are assigned to one or more of 23 such sets (Supplemental Table 2). Figure 4A shows the undirected global significance score in infected young and old mice relative to the same aged uninfected (mock) animals. Higher numbers indicate greater change in the infected state versus uninfected. These results show that non-neurotropic IAV infection alters regulation of brain gene sets in young and old mice, with much greater change in old mice than in young mice (Fig. 4A). The mean ± SD t-statistic at day 3 was 2.12 ± 0.23 versus 3.67 ± 0.54 (*p* < 0.001 by 2-tailed t-test) and at day 7 was 3.09 ± 0.32 versus 3.42 ± 0.40 (*p* = 0.004), respectively, in young and old mice. Directional differences suggested by the directed global significance scores (Fig. 4B) indicated most gene sets were down-regulated after infection in both infection groups and were down-regulated to a greater degree in old than in young mice. These results show that non-neurotropic IAV infection has profound effects on gene expression in the brain. Namely, that regulation of most groups of functionally-related genes trend negative in both young and old mice, and that the changes from the uninfected state are notably more robust in old mice than in young mice.

### Aged mice demonstrate more severe changes in gene expression related to memory loss and cognitive dysfunction by Ingenuity Pathway Analysis

**Fig. 5:**
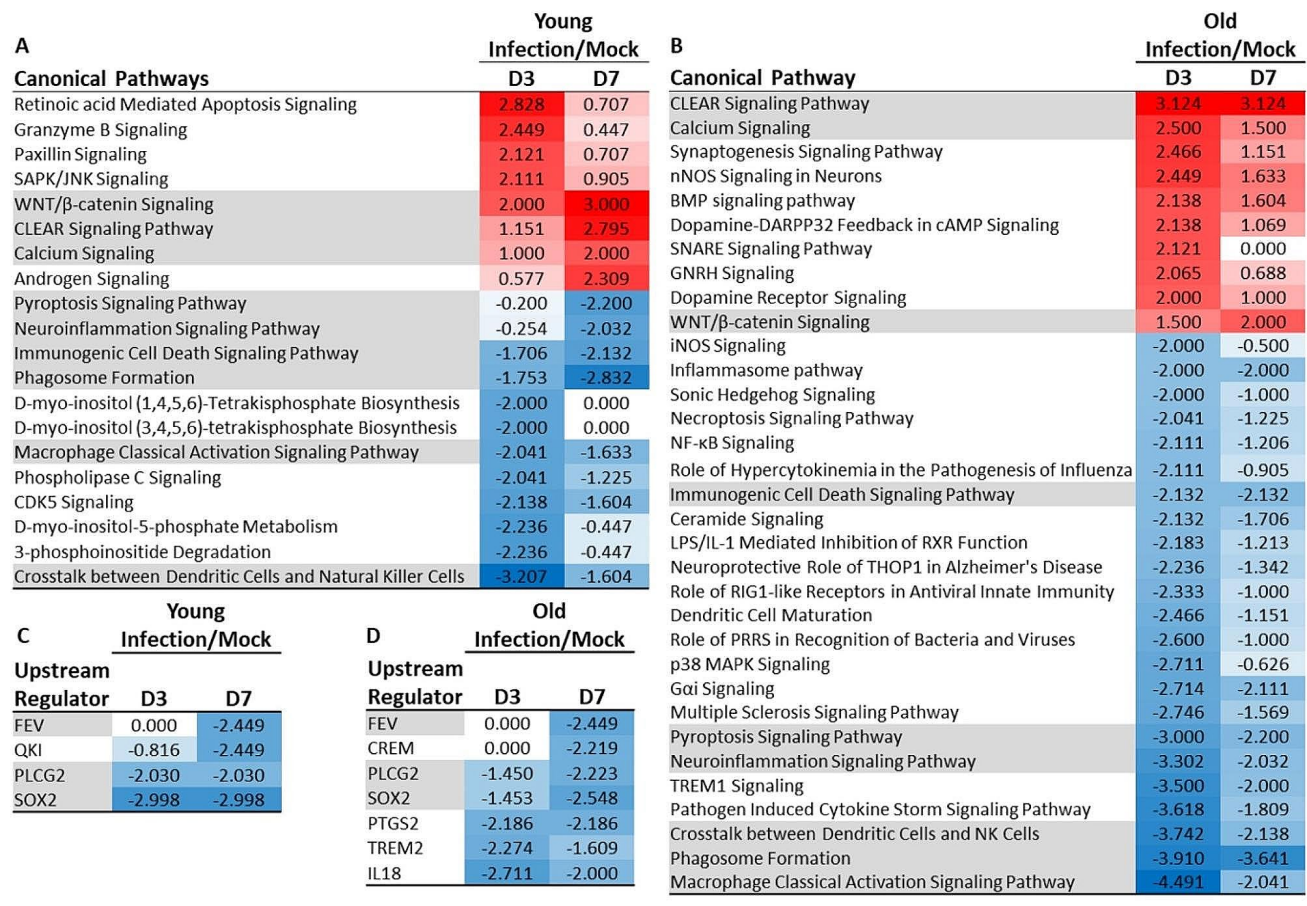
**Non-neurotropic IAV infection induces conserved and distinct signaling pathways and upstream mediators in the brains of young and old mice**. Changes in gene expression in brains of uninfected (mock) young and old mice, and mice 3 and 7 days after IAV infection were quantified by NanoString nSolver 4.0 software and significance in infected mice compared with age-specific uninfected mice was determined by multiple t-test with a Benjamini-Hochberg post-test. Gene lists with associated data were analyzed using Ingenuity Pathway Analysis (Qiagen). Results show z-scores for Canonical Pathways (**A**, **B**) and Upstream Regulators (**C**, **D**) in young mice (**A**, **C**) and old mice (**B**, **D**). Only results with z-score ≤ -2.0 or ≥ 2.0 suggesting inhibition (designated blue and -) or activation (designated red and +), respectively, at either Day 3 or Day 7 p.i. are shown. Shaded rows show pathways identified as inhibited or activated in both young and old mice. Mouse numbers in each group are shown in Fig. 1A

Next, gene expression data were analyzed using IPA (Qiagen) to gain additional insight into changes caused by IAV infection and how responses in the brains of young and old mice differ from each other. Data in Fig. 5 show responses to IAV infection for both age groups compared with their age-specific uninfected (mock) group. The majority of changed pathways in both age groups showed inhibitory effects on the canonical pathway and their imputed upstream regulators. Consistent with results above in Fig. 4, changes were generally more profound in old mice than in young mice. As measured by a Z-score of ≥2 or ≤-2 at either 3 days or 7 days p.i., a total of 20 pathways were changes in young mice and 33 modulated in old mice, with 9 canonical pathways changed in both age groups (Fig. 5A and B). In general, shared pathways were changed in the same direction although with some differences in the magnitude of the Z-score. Analysis of upstream regulators also showed inhibitory effects in both age groups (Fig. 5C, D) as well as a mixture of shared and unique molecules.

**Fig. 6:**
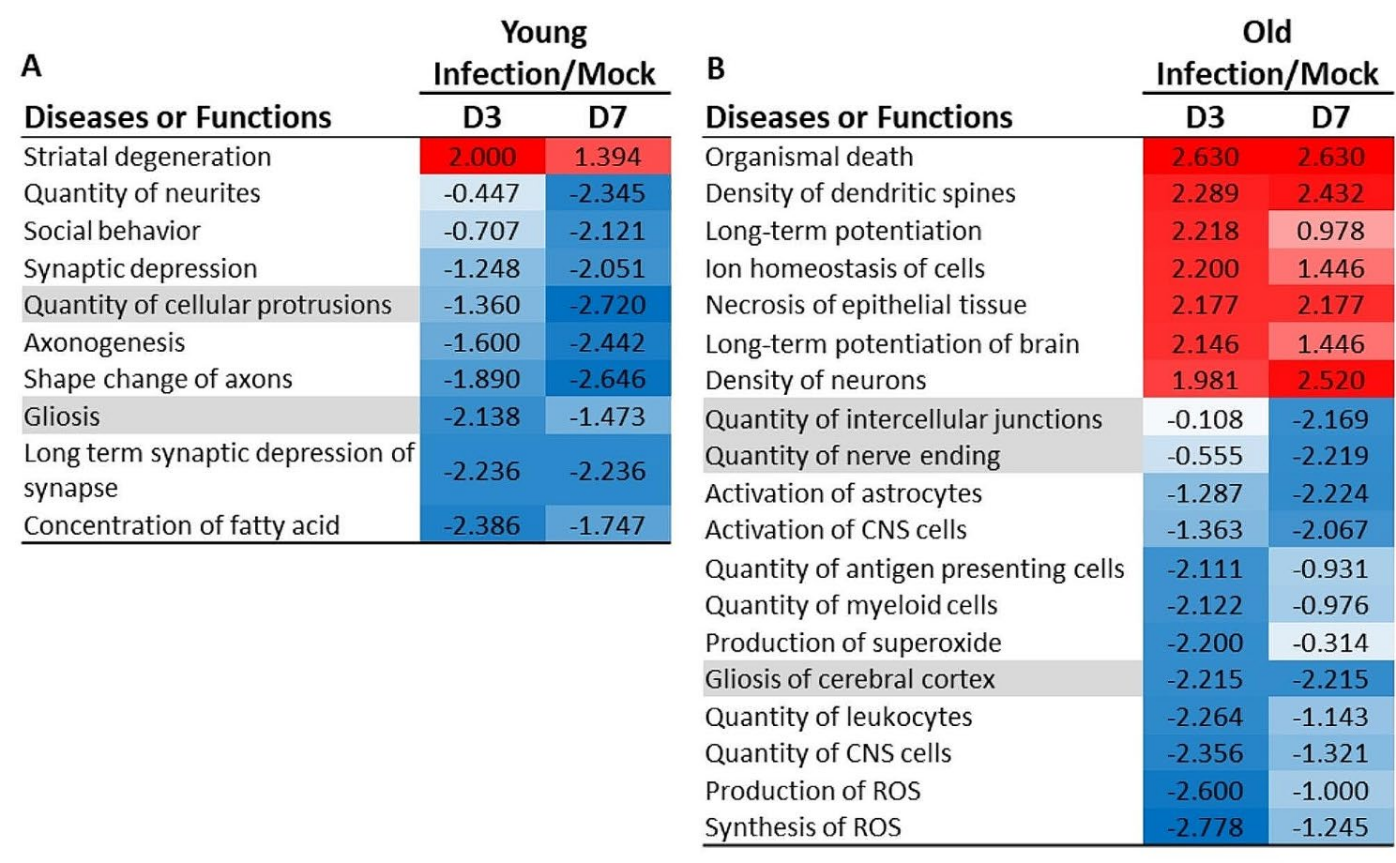
Gene expression changes induced by non-neurotropic IAV infection in young and old mice show different diseases and functions responses. Changes in gene expression in brains of uninfected (mock) young and old mice, and mice 3 and 7 days after IAV infection were quantified by NanoString nSolver 4.0 software and significance in infected mice compared with age-specific uninfected mice was determined by multiple t-test with a Benjamini-Hochberg post-test. Gene lists with associated data were analyzed using Ingenuity Pathway Analysis (Qiagen). Results show Diseases or Functions annotation z-scores in young mice (**A**) and old mice (**B**). Only results with z-score ≤ -2.0 or ≥ 2.0 suggesting inhibition (designated blue and -) or activation (designated red and +), respectively, at either Day 3 or Day 7 p.i. are shown. Shading indicates similar diseases or functions in young and old mice. Mouse numbers in each group are shown in Fig. 1A

In contrast, analysis of IPA Diseases and Functions showed responses that were largely distinct between the age groups (Fig. 6). Although the majority of processes were inhibited in both age groups, this was much more evident in young mice with only 1 of 11 processes identified as potentially activated (Fig. 6A). This compared with 7 of 19 processes potentially activated in old mice (Fig. 6B). Notable results unique to old mice include numerous potentially inhibited pathways related to myeloid cells including cell numbers, activation, and production of reactive oxygen species. Notably, the process with the highest activation score was “Organismal Death”.

**Fig. 7:**
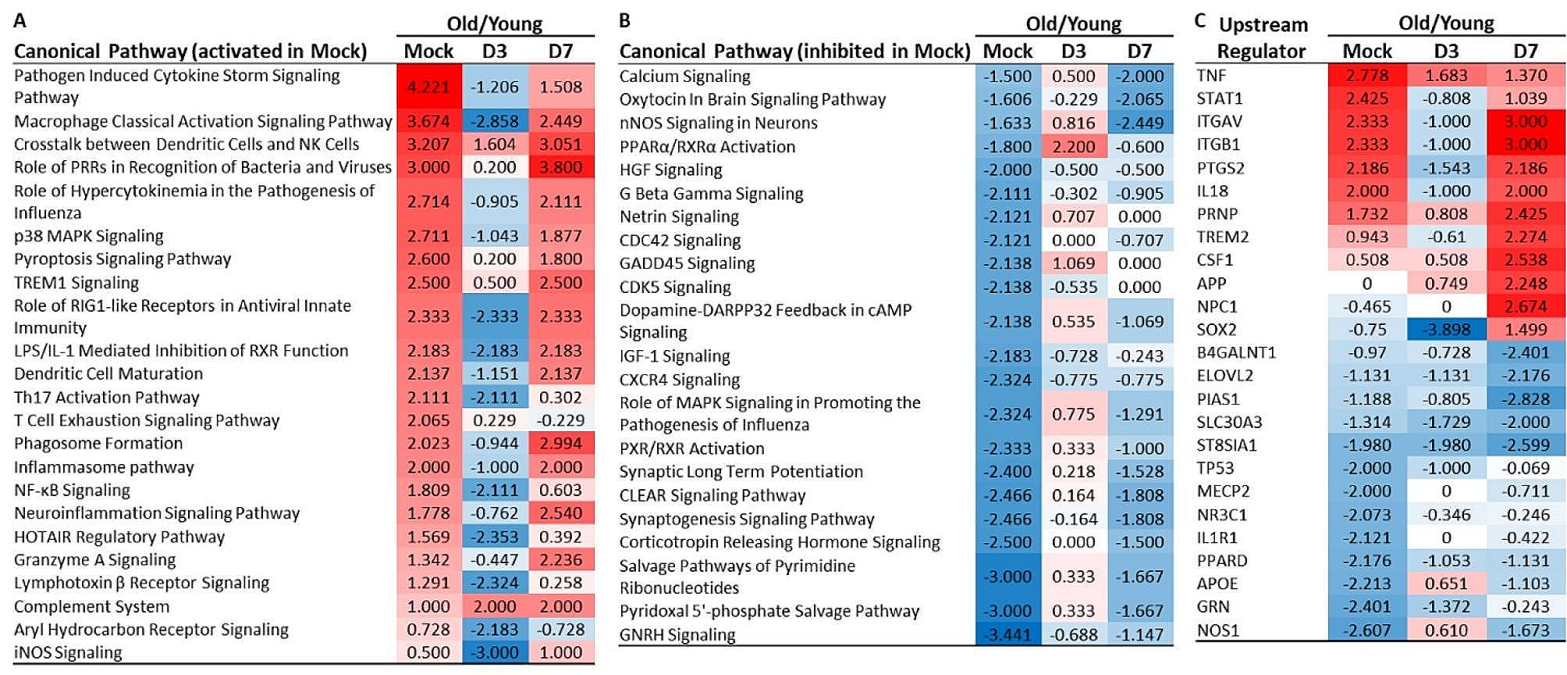
IPA of gene expression in young and old mice without and with IAV infection shows marked differences and responses. Changes in gene expression in brains of uninfected (mock) young and old mice, and mice 3 and 7 days after IAV infection were quantified by NanoString nSolver 4.0 software and significance in infected mice compared with age-specific uninfected mice was determined by multiple t-test with a Benjamini-Hochberg post-test. Gene lists with associated data were analyzed using IPA (Qiagen). Results in (**A**, **B**) show z-scores for Canonical Pathway annotations grouped according to z-scores in uninfected mice indicating activation (**A**, ≥ +2.0, designated as red/+) or inhibition (**B**, ≤ -2.0, designated as blue/-) of the denoted pathways. Upstream Regulators are shown in C. Only pathways or regulators with scores ≥ 2.0 or ≤ -2.0 at any one of the points of measurement (mock, D3, D7) are shown. Mouse numbers in each group are shown in Fig. 1A

**Fig. 8:**
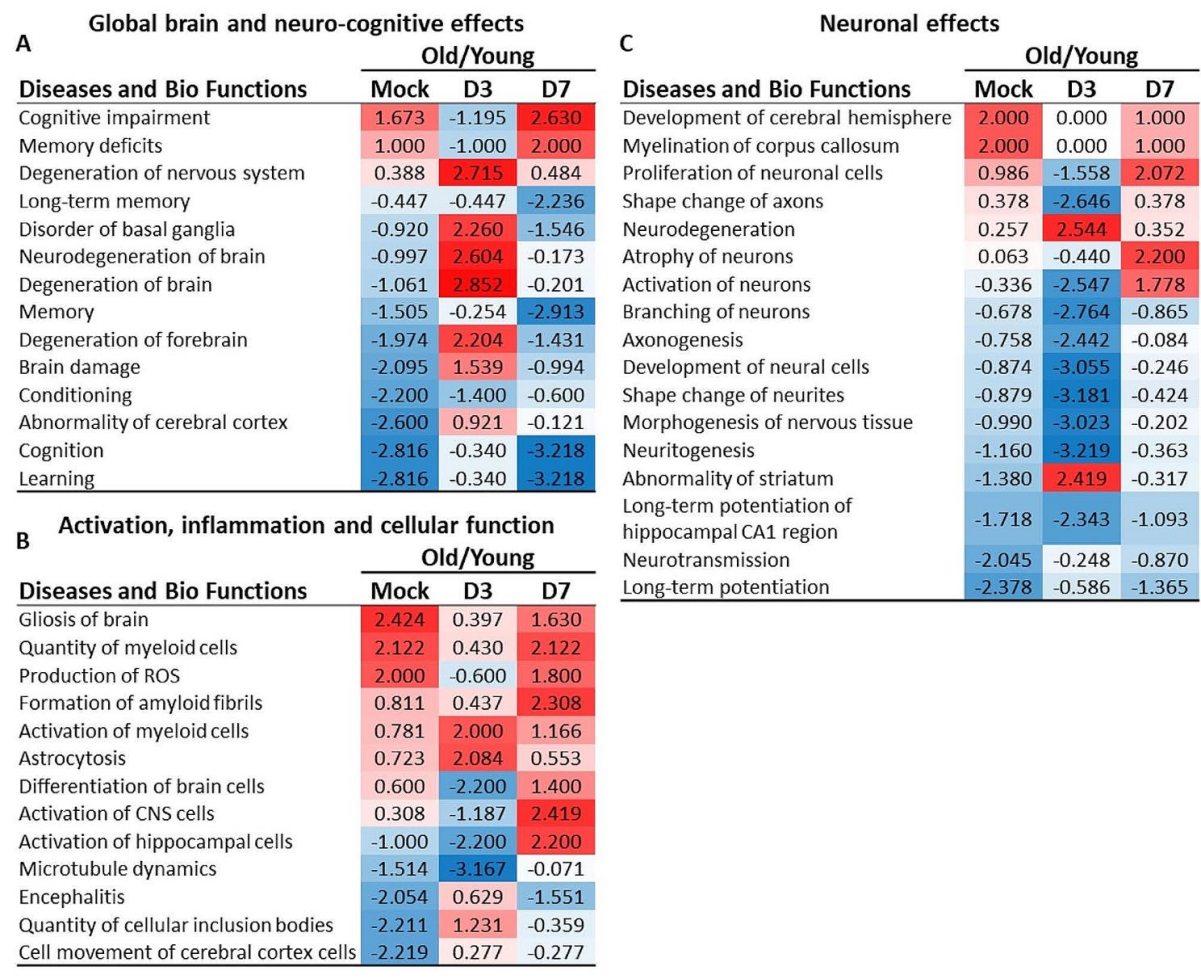
Direct comparison of gene expression in young and old mice suggests non-neurotropic IAV infection worsens neuro-cognitive function in old mice. Changes in gene expression in brains of uninfected (mock) young and old mice, and mice 3 and 7 days after IAV infection were quantified by NanoString nSolver 4.0 software and significance in infected mice compared with age-specific uninfected mice was determined by multiple t-test with a Benjamini-Hochberg post-test. Gene lists with associated data were analyzed using IPA. Results show Diseases or Functions annotation with z-score ≤ -2.0 or ≥ 2.0 suggesting inhibition (designated blue and -) or activation (designated red and +), respectively, in uninfected (mock), or D3 and D7 p.i. old vs. young mice are shown. Mouse numbers in each group are shown in Fig. 1A

Next, we performed direct comparisons of young and old mice (Figs. 7 and 8). As expected, there were notable differences between uninfected mice consistent with excess activation of pro-inflammatory pathways and their associated upstream regulators (Fig. 7A, C). Day 3 post-infection is marked by two general phenomena. The most widespread is that differentially regulated pathways in uninfected (mock) animals are no longer significantly changed (-2.0≤ z scores ≤+ 2.0) in young infected vs. old infected mice. A second observation is that some pathways shift from one pole to the other, e.g. from activation to inhibition as in the Macrophage Classical Activation Signaling Pathway and Role of RIG1-like Receptors in Antiviral Innate Immunity (Fig. 7A) or from non-significantly activated to significantly inhibited, e.g. NF-κB signaling and iNOS signaling (Fig. 7A) and PPARα/RXRα Activation (Fig. 7B). By day 7 p.i., the Z-scores of most pathways were reverting towards their pre-infection state. Over-corrections, as shown in the Neuroinflammation Signaling Pathway and Role of PRRs in Recognition of Bacteria and Viruses and under-corrections (p38 MAPK Signaling) were also observed (Fig. 7A).

Upstream regulators followed a similar pattern to canonical pathways in uninfected mice with evidence of overexpression of pro-inflammatory activity via TNF and IFN (via STAT1) (Fig. 7C). Day 3 also showed reversion towards the mean of nearly all mediators with the exception of the transcription factor sex-determining region Y (SRY)-box 2 (SOX2), which was strongly inhibited. Since only male mice were included in this test, the data is particularly important because this would not be seen in female mice given that they do not have a SRY gene. By day 7, a minority of regulators that were significantly activated or suppressed at the uninfected baseline in old as compared with young mice had returned to the same or similar relative baseline state including ITGAV, ITGB1, PTSG2, and IL18 (Fig. 7C). In contrast, 10 of the 14 regulators with activation scores ≥ 2.0 or ≤ -2.0 in uninfected old mice had not returned to these thresholds by day 7. Additionally, a group of 10 regulators with activation scores (-2.0 ≤ score ≤2.0) in uninfected old vs. young mice had a significant activation or inhibition Z-score by day 7. These include PRNP, TREM2, CSF1, APP, NPC1, B4GALNT1, ELOVL2, PIAS1, SLC30A3, and ST8SIA1. These changes suggest that infection causes a re-ordering, at least temporarily, of inflammatory and metabolic regulators in the brain.

Analysis of Diseases and Biological Functions showed key differences between young and old mice before and during infection (Fig. 8). Remarkably, in uninfected (mock) mice, negative Z scores of brain pathways related to cognition and learning indicated these functions were significantly suppressed in old mice compared to young (Fig. 8A). Key changes at day 3 p.i. included significant Z scores for damaging processes, e.g. degeneration of nervous system and brain (Fig. 8A), and comparative suppression of neuro-preservative functions such as development of neural cells and neurogenesis in old mice (Fig. 8C). By 7 days after IAV infection, differences between old and young mice were even more negative than in uninfected animals in pathways related to cognitive impairment, memory deficits, cognition, and learning (Fig. 8A). This was accompanied by increases in potentially harmful processes such as formation of amyloid fibrils, and activation of CNS cells (Fig. 8B).

## Discussion

Recent studies demonstrate that a variety of non-neurotropic infections in humans significantly increase the risk of serious, post-acute neuro-cognitive complications such as dementia [12–14, 48]. Notably, advancing age has been identified as a key risk factor for dementia following infection severe enough to require hospitalization [12, 14]. Our data show that gene expression in the brain triggered by non-neurotropic IAV infection of C57BL/6 mice could lead to neuronal damage and worsen cognitive function, memory, and learning in 70-week-old mice when compared to 12-week-old mice. These data suggest that older animals express unique gene and pathway changes in responses to IAV infection that might cause neuro-cognitive dysfunction after acute infection.

Experiments reported here compared gene expression in brains of 12-week and 70-week old C57BL/6 mice before and after pulmonary infection with the mouse-adapted PR8 strain of H1N1 IAV. Initial experiments confirmed there was no neuroinvasion and that there was an inflammatory response in the brains of young mice observed by histology, as well as at the mRNA level with upregulation of Arg1, Ifnl, Irf7, Tnf at 3 days p.i. This is in keeping with findings by others that non-neurotropic IAV infection of young mice induces a central inflammatory response [20, 49]. We also confirmed the absence of neuroinvasion in the infected old mice. However, in contrast to findings in young animals, old animals did not upregulate these inflammatory markers despite harboring more virus in the lung at day 3. These results suggested delayed immune responses to influenza associated with aging are also manifested in the brain (Fig. 1B) [7, 50], but the degree to which they might impact development of downstream phenotypic neuro-cognitive complications was not clear.

To study this important question, we analyzed mRNA expression in whole brains of infected and uninfected young and old mice by RT-PCR and nCounts. As expected, baseline differences included up-regulations of genes associated with the inflammatory response in old mice compared with and young mice consisted with previous reports [28, 46]. The baseline levels of pro-inflammatory cytokines, such as Ifnb, Ifnl, IRF7, Il6, in old mice are all higher than those in young mice (Fig. 1B). More striking were baseline differences, whether activated or inhibited, between old and young mice in the IPA® canonical pathways, upstream regulators and diseases and biological functions. Thus, brains of older mice have at baseline a pro-inflammatory environment relative to young mice (Fig. 7A and C). These differences conformed to the concept that the aging brain displays at baseline an enhanced inflammatory/primed milieu compared with younger animals [45, 51–53]. The altered neuroimmune response to IAV in aged individuals could contribute to the disparate cognitive outcomes between young and aged infected patients.

Young and aged mice showed a similar pattern in the direction of regulation of canonical pathways in response to IAV (Figs. 5 and 6). Nonetheless, key differences between young and old mice in the magnitude of these responses were evident. For example, IPA® canonical pathways showed that old mice had greater inhibition of pro-inflammatory signaling pathways compared with young mice at day 3 p.i. This was evident in pathways significantly suppressed by IAV infection in both old and young mouse groups, such as Macrophage Classical Activation. This was also manifested by pathways inhibited in older mice but not in younger mice, e.g. Neuroinflammation, p38MAPK, TREM1, and Pathogen Induced Cytokine Storm. In contrast, the most highly activated pathways in young mice included Granzyme B and Retinoic Acid Mediated Apoptosis signaling. Granzyme B and RIG-I signaling are critical for effective host defenses against IAV, but consistent with the work of others, our results demonstrate they were not activated in old mice [54, 55]. Rather, our data demonstrated that in old mice at day 3 p.i. innate sensing pathways including Role of RIG1-like Receptors in Antiviral Innate Immunity and Role of PRRS in Recognition of Bacteria and Viruses were inhibited compared with uninfected mice.

Direct comparisons of brains from young and old mice at day 3 p.i. also showed that the general over-expression of proinflammatory pathways in uninfected (mock) old mice compared with young mice was no longer evident. For example, Z-scores of inflammatory canonical pathways, e.g. Macrophage Classical Activation Signaling and Role of RIG1-like Receptors in Antiviral Innate Immunity suggested they were inhibited (Fig. 7A and B Day 3). In addition, at 3 days p.i., there was inhibition of SOX2 as an upstream regulator in infected old mice compared with infected young mice (Fig. 7C). SOX2 is a transcription factor with a critical role in maintaining multi-potency of neural precursor cells, including in the adult hippocampus [56, 57]. Interestingly, its putative activity as an upstream regulator was also inhibited by IAV infection in mice of both ages compared to age-specific uninfected mice (Fig. 5C, D). These results suggest that non-neuroinvasive IAV infection could have negative effects on neurogenesis in adults irrespective of age particularly in old adults.

By 7 day p.i., many of the differentially regulated pathways, upstream regulators, and disease processes in old mice compared with young mice were returning towards the uninfected (mock) state. However, there were exceptions which are interesting in the context of the findings that non-neurotropic IAV induces neuronal damage and cognitive impairment in adult mice [18, 19, 49]. IPA analysis identified neurocognitive functions that were impaired (e.g. Cognition, Memory, Learning, Long-term memory) or over-expressed (e.g. Cognitive impairment and Memory deficits) to a greater degree in old mice than in young mice by 7 days after infection.

**Fig. 9:**
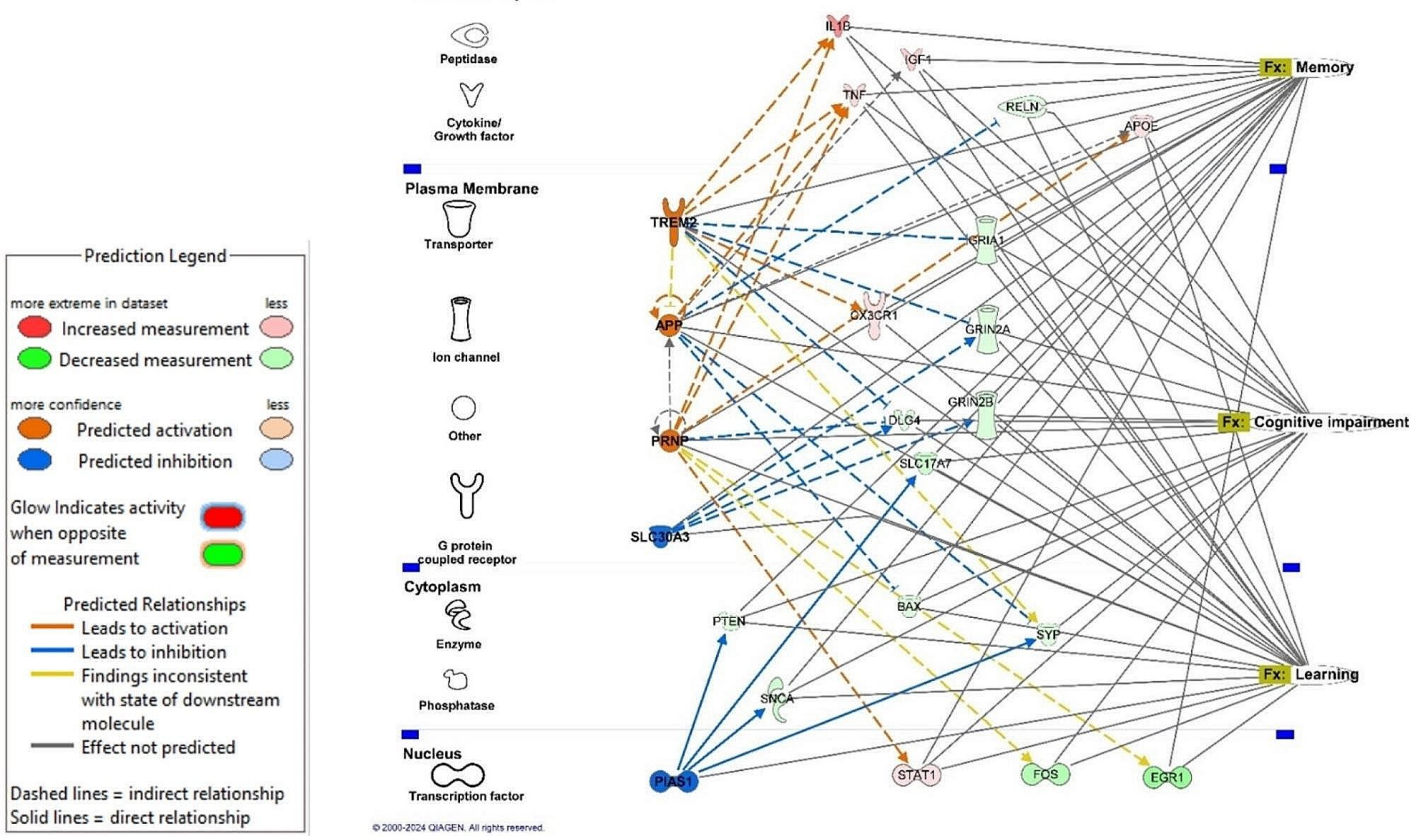
Model for interaction between predicted upstream regulators, array molecules, and downstream neurocognitive effects of non-neuroinvasive IAV infection. Changes in gene expression in brains of uninfected (mock) young and old mice, and mice 3 and 7 days after IAV infection were quantified by NanoString nSolver 4.0 software and significance in infected mice compared with age-specific uninfected mice was determined by multiple t-test with a Benjamini-Hochberg post-test. Gene lists with associated data were analyzed using IPA. Diagram shows interactions between upstream regulators (TREM2, APP, PRNP, SLC30A3, and PIAS1) differentially regulated (Z-scores ≤-2.0 or ≥ + 2.0) between old and young mice at 7 days p.i. array molecules in their pathways, and key downstream biological functions of Cognitive impairment, Memory, and Learning. Figure constructed in IPA Path Designer. Legends showing predicted activations and interactions and describing shapes representing various types of molecules are shown

Collectively, these data suggest that non-neurotropic IAV infection triggers changes in multiple pathways in the brain that could worsen neurocognitive function in old animals to a greater extent than those found in young ones. To illustrate this model, we used IPA to identify putative connections between upstream regulators that were differentially regulated in old compared with young mice at 7 days p.i., i.e. Z-score ≤ -2.0 or ≥ 2.0, and the downstream biological functions of Cognitive impairment, Memory, and Learning (Fig. 9). This showed direct connections between TREM2, APP, PRNP, SLC30A3, and PIAS1, their downstream molecules, and key neurocognitive pathology. Several upstream regulators had higher or lower expression in old mice at 7 days after infection than in uninfected (mock) animals and are directly or indirectly linked to neurocognitive pathology (Fig. 9). Those with increased expression included APP (Amyloid β Precursor Protein), TREM2 Triggering Receptor Expressed on Myeloid Cells 2, and PRNP (Prion Protein), whereas PIAS1 (Protein Inhibitor of Activated STAT1) and SLC30A3 (Solute Carrier Family 30 member 3) were down-regulated compared to uninfected (mock). Knowledge of upstream mediators is useful for forming hypotheses for experimental testing regarding how IAV infection triggers negative neurocognitive consequences. Two examples include APP and PIAS1. The protein encoded by APP is essential to the pathophysiology of Alzheimer’s Disease [58]. There are also data indicating that this protein, or fragments of it, have anti-viral properties against IAV as well as other viruses [59, 60]. Thus, it could be part of a host defense mechanism that has untoward consequences in the brain. PIAS1, an anti-inflammatory molecule that down-regulates type I IFN signaling, has been shown to impair special learning in a rat model [61, 62]. Decreased expression in old mice than in young mice at day 7 p.i. found here would be predicted to produce negative effects on neurocognitive function. These results indicate that changes in gene expression in the brain during the first week of IAV infection could underlie the finding that old individuals are at a greater risk than young ones for developing pathologic neuro-cognitive sequelae after peripheral infections [12, 14].

Our data show that brains of old animals have unique gene and pathway changes in responses to IAV infection that might cause neuro-cognitive dysfunction after acute infection. How these age-enhanced negative effects of IAV infection on brain function relate to age-related differences in the brain innate immune response is unknown and will require further investigation. The goal of the current report was to study the effects of aging on brain gene expression and innate immune responses to IAV infection. The study focused on male mice and used the smallest sample size needed to ensure adequate statistical power in order to reduce the number of animals used both in compliance with IACUC guidelines, and to conserve resources. Future investigations will be expanded to evaluate sex as a biological variable by using female mice in subsequent studies.

## Conclusions

These data suggest the worse learning and cognitive performance that exists at baseline in old mice is further worsened by IAV infection, similar to that seen in aged human patients. Early events in the brain triggered by IAV infection portend downstream neurocognitive pathology in older adults.

### Electronic supplementary material

Below is the link to the electronic supplementary material.

Supplementary Material 1

Supplementary Material 2

Supplementary Material 3

Supplementary Material 4

Supplementary Material 5

## Data Availability

The original contributions presented in the study are included in the article/Supplementary Material. Further inquiries can be directed to the corresponding authors.
