# Supplementary material for: Influenza virus infection exacerbates gene expression related to neurocognitive dysfunction in brains of old mice
Source: Immun Ageing. 2024 Jun 21;21:39. doi: 10.1186/s12979-024-00447-y (PMC11191167; doi:10.1186/s12979-024-00447-y)
Supplement: Supplementary file 1 — Supplementary Material 1 [file 12979_2024_447_MOESM1_ESM.docx]

Supplemental Table 1.

List of mouse primers used in RT-PCR.

| **Gene** | **Forward primer (5’-3’)** | **Reverse primer (5’-3’)** |
| --- | --- | --- |
| BDNF | TGCAGGGGCATAGACAAAAGG | CTTATGAATCGCCAGCCAATTCTC |
| NRGN | TCCAAGCCAGACGACGATATT | CACACTCTCCGCTCTTTATCTTC |
| GRIN2B | CAGCAAAGCTCGTTCCCAAAA | GTCAGTCTCGTTCATGGCTAC |
| SNCA | ATACGGTGGAATACCAGAAAGGG | CATCCATGTCGAGGTCATAGTG |
| CASP1 | ACAAGGCACGGGACCTATG | TCCCAGTCAGTCCTGGAAATG |
| RALA | ATGTACGACGAGTTTGTAGAGGA | CCCGCTGTATCTAAGATGTCGAT |
| SYP | TTCAGGACTCAACACCTCGG | CGCATCGCCGTAGGCATCGC |
| M1 | ATGAGCCTTCTAACCGAGGTC | TGGACAAAACGTCTACGCTGCAG |
| ifnb | CCATCAACTATAAGCAGCTCCAGC | CCACCATCCAGGCGTAGCTGTTG |
| β-actin | CAGAAGGACTCCTATGTGGGTG | GGATCTTCATGAGGTAGTCTGTC |
| IRF7 | CAGCGAGTGCTGTTTGGAGAC | AAGTTCGTACACCTTATGCGG |
| IL-6 | CCGGAGAGGAGACTTCACAG | GGTACTCCAGAAGACCAGAGG |
| ifnl | AGCTGCAGGCCTTCAAAAAG | TGGGAGTGAATGTGGCTCAG |
| TNFα | GCCCAAGGCGCCACATCTCC | CCACTTGGTGGTTTGCTACG |
| Arg1 | GGAAGACAGCAGAGGAGGTG | TATGGTTACCCTCCCGTTGA |
| Nos2 | CTGCTGGTGGTGACAAGCACATTT | ATGTCATGAGCAAAGGCGCAGAAC |
