# Supplementary material for: Influenza virus infection exacerbates gene expression related to neurocognitive dysfunction in brains of old mice
Source: Immun Ageing. 2024 Jun 21;21:39. doi: 10.1186/s12979-024-00447-y (PMC11191167; doi:10.1186/s12979-024-00447-y)
Supplement: Supplementary file 5 — Supplementary Material 5 [file 12979_2024_447_MOESM5_ESM.docx]

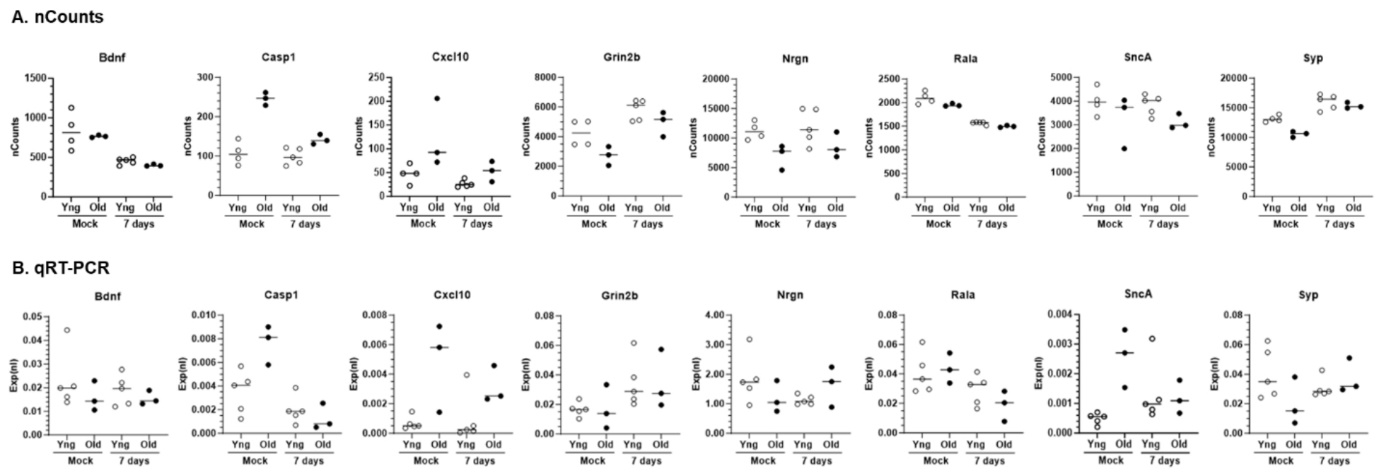


**Supplemental Fig. 1. Comparison of gene expression in the brains of young and old mice measured by nCount or by qPCR**. Gene expression in whole brains of uninfected young and old mice (Mock), and in mice 7 days after infection with 200 PFU IAV/mouse were measured by nCounts using the NanoString Neuroinflammation Array (A) or by qRT-PCR normalized to β-actin (B). Mouse numbers in each group are shown in Fig. 1A.
